# The Long-Term Use of Methotrexate Monotherapy as a Steroid-Sparing Agent for Patients With Pulmonary Sarcoidosis

**DOI:** 10.7759/cureus.17509

**Published:** 2021-08-27

**Authors:** Ahmad F Baiyasi, Najeebullah Bangash

**Affiliations:** 1 Pharmacology and Therapeutics, Wayne State University School of Medicine, Detroit, USA; 2 General Internal Medicine, Beaumont Hospital, Dearborn, USA

**Keywords:** clinical decision-making, steroid-induced diabetes, extra pulmonary manifestations of sarcoidosis, oral methotrexate, critical appraisal

## Abstract

This report aims to determine the benefits of long-term use of methotrexate as a steroid-sparing agent for a patient with pulmonary sarcoidosis who cannot tolerate the glycemic effects of steroids. A search for articles using the medical subject heading terms “methotrexate” and “sarcoidosis” on PubMed involving clinical trials evaluating the therapeutic effect and toxic profile of methotrexate as a steroid-sparing agent in treating pulmonary and/or extrapulmonary symptoms of sarcoidosis was included. The literature review indicates that methotrexate is an alternative treatment for sarcoidosis, allowing the patient to avoid the long-term side effects of steroids while achieving similar rates of treatment and remission. Patients in several research studies were able to taper their steroid dosage over time when methotrexate was concurrently prescribed for the treatment of pulmonary sarcoidosis. The critical appraisal of one of the studies reviewed indicates methotrexate as a single agent in treating patients with chronic progressive pulmonary sarcoidosis, serving as a steroid alternative. Methotrexate was safe and effective for a duration of 6-24 months, with patients experiencing definite improvements in pulmonary function tests. However, not all patients in this open prospective, real-life, single-center trial demonstrated improvement. Thus, a review of the current literature is often necessary to help guide clinical decision-making for patients with chronic diseases like sarcoidosis and to determine patient characteristics of methotrexate responders.

## Introduction

Sarcoidosis is a multisystemic disease characterized by noninfectious, granulomatous inflammation throughout multiple organ systems, notably the lymph nodes and lungs. Symptoms can include a combination of chronic dry cough, lymphadenopathy, systemic signs of inflammation such as weight loss and fatigue, arthralgias, and even cutaneous manifestations like erythema nodosum. Treatment may not be required in asymptomatic cases, but steroids are typically used to treat flare-ups of the disease. They can also be used for long-term therapy with subsequent taper, typically over one year. However, steroids are contraindicated in patients prone to insulin resistance and the development of diabetes. Other therapies, including methotrexate, have also been used for both pulmonary and extrapulmonary manifestations of sarcoidosis. Methotrexate is typically used as a steroid-sparing agent in a variety of immunological conditions. For patients who develop adverse effects require a prolonged course or have contraindications to steroids, methotrexate may be a viable alternative [1].

## Case presentation

The patient is a 35-year-old African American who will be identified by the alias Rose Jones. She has a primary diagnosis of systemic sarcoidosis. She was diagnosed one year ago after presenting to her primary care physician with a complaint of progressive dyspnea upon exertion for the previous several months and intermittent arthralgia in her wrists and ankles. A chest x-ray revealed bilateral pulmonary nodules, hilar lymphadenopathy, and minor interstitial fibrosis. Pulmonary function tests (PFTs) revealed a 17% decrease in total lung and vital capacity. A bronchial biopsy demonstrated non-necrotizing granulomas, confirming the diagnosis of sarcoidosis. She was started on prednisolone 20 mg orally upon initial presentation but developed high fasting serum glucose levels four months later and subsequently developed type 2 diabetes mellitus. Her BMI prior to treatment was 30 kg/m^2^. Prednisolone was tapered and discontinued. She was started on metformin 500 mg orally once weekly. In addition, she was prescribed ibuprofen 400 mg orally on a per-needed basis for her exacerbation of intermittent joint flares. Her dyspnea returned and progressed, and thus she was started on methotrexate 15 mg orally once a week a few months later. She was also prescribed 5 mg oral folic acid to be taken daily except on the day of methotrexate use. She had been taking methotrexate for eight months. Her recent visit was her typical bi-monthly follow-up on her symptoms. Blood was also drawn from the patient to monitor the toxic profile of methotrexate, which indicated liver function tests and hemoglobin within the reference ranges. She has not experienced any shortness of breath in the past six months, nor any anemia or other methotrexate-induced toxicities. Her last arthralgia flare was five months prior. She inquires about the efficacy and safety of her continuous use of methotrexate. She is curious about the safety of the long-term usage of methotrexate as she feels worried about the labs she has consistently done every two months. However, she did not have any adverse effects from methotrexate, any abnormal labs, new symptoms, or physical exam findings suggestive of methotrexate toxicity. She had tolerated the medication well overall.

## Discussion

Literature review

This paper is a critical appraisal in which pertinent literature relevant to this patient's clinical context is first explored, with the most clinically relevant study appraised and analyzed in more detail in order to determine the viability of methotrexate monotherapy in this patient with sarcoidosis.

A search for articles using the medical subject heading terms “methotrexate” and “sarcoidosis” resulted in 502 results on PubMed. Modifying the search criteria to include only clinical trials and randomized controlled trials resulted in 16 results. A review of the articles’ citations was also used in order to find several more clinical trials not included in the modified search results. Only clinical trials evaluating the therapeutic effect and toxic profile of methotrexate as a steroid-sparing agent in treating pulmonary and/or extrapulmonary symptoms of sarcoidosis were included.

A double-blind, randomized controlled trial was performed on 24 patients diagnosed with symptomatic sarcoidosis treated with prednisone within the first year of initial diagnosis [1]. The purpose of this study was to see if methotrexate can be used as a steroid-sparing agent in the first year of steroid therapy for patients with sarcoidosis. Patients were assigned to either receive methotrexate or a placebo for one year. Prednisone was tapered on a predetermined schedule monthly. Only 15 participants received a minimum of six months of therapy and were thus used for further statistical evaluation. It was determined that there was a reduction in prednisone taken per day for both groups [1]. The methotrexate group tapered the amount of prednisone a greater amount when compared to the placebo group (p < 0.01) and also gained less weight. Unlike the previous studies mentioned, there was no difference in the toxic profile between methotrexate and placebo. The study concluded that methotrexate can be used as a steroid-sparing treatment in patients with acute sarcoidosis. The major strength of this study was its design, which unlike the previous study discussed employed a blinded control and placebo group. The study indicates that methotrexate users gained less weight, which is a positive effect in Ms. Jones’s case. However, this study is a weaker body of evidence for methotrexate’s ability to treat sarcoidosis in patients unable to tolerate corticosteroids and would not serve to clinically guide Ms. Jones’s treatment plan. The original sample size severely limits the statistical power of the study; the attrition bias and subsequent evaluation of only 15 patients make the study even less powerful. Further, six months may not have been an adequate time to evaluate for methotrexate’s toxic profile or efficacy. The fact that methotrexate and the placebo had the same toxic profile may point to this. Literature commonly states that a period of six months is the limited time to evaluate for methotrexate’s efficacy, and thus the improvements noted in the control group may be confounded due to the spontaneous remissions commonly found in sarcoidosis [1].

A randomized controlled trial was performed by assigning 12 patients with symptomatic pulmonary sarcoidosis into two treatment groups, a methotrexate group and a corticosteroid group [2]. Baseline PFTs and bronchoscopies with bronchoalveolar lavage were conducted on patients before and after a minimum of six months of treatment of either methotrexate 10 mg oral or prednisone. It was found that both treatment groups had improvements in pulmonary vital capacity and fewer lymphocytes in the lavage [2]. Macrophages found in the lavage were also less likely to release tumor necrosis factor and hydrogen peroxide in both treatment groups, which resulted in less interstitial inflammation in the lunch parenchyma. The study concluded that both treatment options were successful in reducing the inflammatory cells found in the lavage and increased clinical pulmonary functioning. The study directly compared the effects of methotrexate monotherapy to corticosteroid monotherapy, which differs from the previous studies that examined methotrexate's ability to act as a steroid-sparing agent. The direct comparison allows for further enhancement of methotrexate’s ability to serve as monotherapy, such as in the case of Ms. Jones, who underwent a repeat bronchoscopy after eight months of taking methotrexate, which demonstrated reduced granulomatous inflammation. The utilization of a control group and the specificity in quantitative, measurable improvements also enhance this study’s conclusion. However, the study was not randomized, nor was it blinded, and some patients in the methotrexate group also received low-dose prednisone, which significantly affects the study’s internal validity. The sample size is also small, which limits its statistical power. Joint pain, Ms. Jones’s extrapulmonary manifestation of sarcoidosis, was also not accounted for in this study.

A systematic review conducted by Baughman et al. analyzed the use of methotrexate in chronic sarcoidosis patients, who are defined as someone with clinical manifestations of the disease for two or more years. Chronic sarcoidosis patients often seek pharmacological alternatives to corticosteroids due to the adverse effects that result from long-term use. A systematic review of case studies involving patients with cutaneous and arthralgic sarcoidosis treated with prednisone saw significant improvement in symptoms after starting methotrexate therapy [3]. Patients with pulmonary signs of sarcoidosis saw a 50% change in improvement. In 50 patients treated with methotrexate over the course of two years, the overall response rate was 66%; 209 patients treated with methotrexate for a minimum duration of six months found 52% of patients to be in remission. The study concluded that patients with chronic sarcoidosis refractory to systemic corticosteroids or seeking steroid-sparing agents due to experienced side effects are candidates for methotrexate. If methotrexate is to be used, treatment is initiated for a minimum of two years; thereafter revaluation with liver biopsy and withholding methotrexate for a duration of two months will help determine if the patient clinically deteriorates and should restart methotrexate therapy. Monitoring the patient’s white blood cell count and liver enzymes is important during regular follow-up visits, a routine assessment found in the previously discussed studies. This systematic review indicated that renal function is crucial, as methotrexate is renally eliminated, and raises the concern of concomitant use of nonsteroidal anti-inflammatory agents, necessitating even closer monitoring of renal function. The collective conclusion indicated that methotrexate was an acceptable alternative for patients with chronic or refractory sarcoidosis. The systematic review exhibited strength in further qualifying methotrexate as a steroid-sparing regimen in patients with sarcoidosis with multiple trials and case studies. Some of the studies used in this review were individually analyzed in the previous paragraphs under the literature review. Further, this review focused on the use of methotrexate over a two-year period; Ms. Jones was using methotrexate for one year, and this study can help determine the next steps in the management of her chronic sarcoidosis with methotrexate in the year to come. Ms. Jones is mainly concerned with her dyspnea and arthralgia, which directly relates back to the reviews’ findings. Ms. Jones currently takes ibuprofen for intermittent joint flares, and this study highlighted the importance of monitoring renal function when using methotrexate, which is renally eliminated. This potential pharmacological interaction is an important consideration when holistically reviewing the use of methotrexate in Ms. Jones’s clinical situation.

A prospective, single-center open trial conducted by Goljan-Geremek et al. on 50 patients with biopsy-diagnosed sarcoidosis investigated the therapeutic efficacy and safety of methotrexate monotherapy. The study concluded that methotrexate monotherapy was a viable alternative to steroid therapy in patients with progressive pulmonary sarcoidosis who could not or were not willing to tolerate long-term steroid therapy [4]. Due to the study’s inclusion and exclusion criteria in the determination of eligible participants, the objective and quantifiable clinical parameters used to evaluate for treatment efficacy, the two-year duration for measurement of results, and the baseline comparison of pulmonary function both before and after methotrexate monotherapy, this study was selected for critical appraisal. This high-quality prospective cohort study with a patient-oriented design has an evidence level of 2 and a B level strength of recommendation per the Strengths of Recommendation Taxonomy [5].

Critical appraisal

The study design of the Goljan-Geremek et al. trial consisted of a prospective, single-center cohort open-label project [4]. A randomized double-blinded placebo control trial was not implemented in this case because the chosen patients were the patients experiencing chronic, symptomatic pulmonary sarcoidosis refractory to steroid therapy or who could not tolerate the adverse effects of steroids. Because this study investigated the ability of methotrexate to serve as a single agent for pulmonary sarcoidosis, it would have been unethical to assign symptomatic patients into a placebo-treated group, essentially leaving them untreated for the duration of the study. This sample population is similar to Ms. Jones, who has progressive pulmonary sarcoidosis and who could not tolerate steroid therapy due to its hyperglycemic effects. The patients were recruited from the Respiratory Department of the National Tuberculosis and Lung Diseases Research Institute, and the decision to recruit them for the study was determined by a team of clinicians who had been responsible for the care of these patients up to this point. The patients were recruited over a time period extending from 2004 to 2013, indicative of a consecutive recruitment process. This helped to increase the insight regarding the eligible number of patients and increased the ability of researchers to calculate response rates using clinical information. It also decreased sampling bias as it includes a temporal spectrum of the available subjects. The fact that the study’s sample consists of patients who were recruited after a holistic review of their clinical standing rather than a random sample of volunteers elucidates one of this study’s strengths as it ensures the internal consistency of its participants. The study excluded participants who were currently taking steroid therapy, whether for sarcoidosis or some other reason.

The inclusion and exclusion criteria for all participants were well-defined and delineated clinical criteria necessary to be filled by a subject to partake in this study. Prior to the start of the trial, two or more measurements of each of the following was required: a functional decline in breathing in after six-minute walk test, a 10% decrease or more in forced vital capacity, total lung capacity, and forced expiratory volume in one second, or a 10% decrease in diffusion capacity. Radiographic evidence of fibrotic parenchymal disease was also required. Spontaneous remission is, unfortunately, a confounding variable due to the nature of sarcoidosis. Ensuring that all patients fulfilled the definition of chronic pulmonary sarcoidosis as defined by the World Association of Sarcoidosis and Other Granulomatous diseases (WASOG) Task Force as a demonstration of progressive physiological or radiological damage helped to decrease the confounding issue. Alternatively, because chronic progressive sarcoidosis is based on progressive radiological damage, decreased exercise capacity, and decreased pulmonary function, this group of patients will inevitably be considered a heterogeneous sample, which may hinder the internal validity of this study. It makes the determination of the clinical effectiveness of methotrexate equivocal. Ms. Jones fulfills this study’s inclusion criteria, with a 17% decrease in PFTs, and radiographic imaging significant for pulmonary fibrosis, enhancing this study’s external validity and clinical decision-making guidance for our patient’s population, patients with progressive pulmonary sarcoidosis unable to tolerate steroid therapy.

The study protocol consisted of a six-month period consisting of no therapy, with baseline measurements of pulmonary function and routine laboratory tests in order to evaluate contraindications to methotrexate prior to treatment. Patients after this six-month period were evaluated for pulmonary function once more and showed significant deterioration in lung measurements, justifying their participation in the study. Patients were then administered methotrexate 10-15 mg by mouth (PO) once weekly and 5 mg folic acid PO every day for a planned duration of 24 months. A weak point in the study’s design is that there were no strict criteria to justify administering either 10 mg or 15 mg at the start of the study, which makes it difficult to ascertain the effective dose relation. The evaluation was done every six months to determine if treatment would be withdrawn due to progressive pulmonary decline, intolerance of therapy, or stabilization of disease. Possible toxicity was monitored every four to six weeks, and the pulmonary function was evaluated every six months, a routine assessment found across multiple studies mentioned in this paper. Another weakness of design can be attributed to the lack of a precise end date for therapy, which would allow for a more reliable calculation of the relationship between the duration of therapy and dose with clinical improvement. Different patients could experience clinically improving effects of methotrexate at different times, which would call for further investigation. This protocol allows for patients to serve as their own controls, a major strength in the study design.

Seventy-six patients were initially recruited for the study, and 50 patients met inclusion and did not meet exclusion criteria. Forty-nine patients were used for statistical analysis due to one noncompliance. Although the statistical power of the study is limited and limits the generalizability to larger populations, the lack of patient attrition significantly decreases attrition bias. Patients were termed methotrexate responders if they experienced a 10 percent improvement in either forced expiratory volume in one second (FEV1), forced vital capacity (FVC), or total lung capacity (TLC), or a 15% improvement in diffusing capacity of the lungs for carbon monoxide (DLCO) from the time of initiation of methotrexate to the time of termination of therapy. These are clinically relevant outcomes and universally acceptable and applicable tests on patients with pulmonary function decline regardless of etiology. As such, this high-quality prospective cohort study with a patient-oriented design has an evidence level of two and a B level strength of recommendation per the Strengths of Recommendation Taxonomy. All cases were included in statistical analyses, regardless of the duration of treatment, to ascertain the number of patients responsive to the treatment.

Thirty-one patients received 10 mg of methotrexate, and 18 patients received 15 mg. Eighteen patients in the 10 mg group were classified as nonresponders, and six patients in the 15 mg group were classified as nonresponders. The retrospective analysis confirmed which patient fulfilled the objective measures to be qualified as responders to therapy, and an intention to treat analysis was performed. Fourteen patients received 24 months of methotrexate therapy, and 16 patients terminated therapy after six months for reasons mentioned previously. Nineteen patients received more than six and less than 24 months of therapy due to stabilization of disease or subjective intolerance of methotrexate. Patients receiving the 15 mg dose of methotrexate experienced a statistically decreased DLCO of more than 10% compared to their baseline values before the onset of the study when compared to patients in the 10 mg group. Ms. Jones is taking 15 mg of methotrexate, and thus this finding helps to clinically assure that she is on a beneficial regimen to treat her dyspnea. The effect size of DLCO between the responder and nonresponder group was 0.21, calculated using Cohen’s delta. No significant increase in pulmonary function testing relative to baseline measurements was found in the cohort in its entirety at the end of the study.

Generalizing the results of this study to Ms. Jones's improvement in her dyspnea after starting methotrexate, it should be further explored whether Ms. Jones’s pulmonary improvement involves a decrease in her DLCO rather than in an increase in her PFTs. It was determined that methotrexate responders on average received significantly higher amounts of methotrexate for longer periods of time; however, these patients had significantly lower TLC and FVC values at baseline when compared to non-methotrexate responders. Although no other significant baseline differences were observed between responders and nonresponders, this difference in baseline affects the generalizability of results to patients with pulmonary sarcoidosis. Eleven patients terminated treatment due to adverse effects of methotrexate, including gastrointestinal discomfort. The most frequently reported adverse effects were hepatic in origin, including an elevation in liver enzymes and bilirubin. However, after discontinuation of therapy, liver enzymes and function recovered, and no hematological disorders were diagnosed. Regarding safety parameters, no significant changes were noted in the cohort as a whole. Alanine aminotransferase (ALT) values were significantly increased post-treatment, but the mean value was still within normal limits. Routine assessment of Ms. Jones’s lab values is thus considered a standard protocol in accordance with this study and the aforementioned studies noted in the literature review. The study concluded that methotrexate monotherapy was effective as a steroid-sparing treatment in patients with pulmonary sarcoidosis, which can help guide Ms. Jones's clinical situation. The effect of methotrexate on sarcoidosis-associated joint pain was not assessed, and extrapulmonary manifestation requires more research as evidenced by the lack of such information in all studies noted in this paper.

Clinical application

Literature review indicates the steroid-sparing benefits of methotrexate. Patients in several research studies were able to taper their steroid dosage over time when methotrexate was concurrently prescribed for the treatment of pulmonary sarcoidosis. The critical appraisal of Goljan-Geremek et al. indicates methotrexate’s use as monotherapy, which is Ms. Jones’s current pharmacological regimen, enhancing the study’s external validity and clinical applicability to this patient [4]. Ms. Jones had progressive pulmonary deterioration over several months when her steroid therapy was discontinued, which justifies the use of methotrexate in place of steroid therapy; if actual PFT were measured at the onset of steroid withdrawal and six months later, she may have very well fulfilled the inclusion criteria of the study. The study’s design and data stratification regarding the dosage and duration of methotrexate use on pulmonary function strengthen the internal validity and qualify the conclusion reached. The longest duration of methotrexate use was two years, which would mean Ms. Jones may need further evaluation regarding the use of methotrexate within the next 16 months of use. She has four children and had a hysterectomy, so the teratogenic effects associated with methotrexate are not relevant.

However, Goljan-Geremek et al. validated the concomitant use of folic acid with methotrexate, which should be considered for Ms. Jones, regardless of her reproductive functioning [4]. This would protect her from hematological abnormalities, a common toxic effect of methotrexate. She has not had an elevation of liver enzymes thus far and may require a liver biopsy to ensure hepatic functioning, but she should continue with routine laboratory testing to evaluate for any toxicity. The onset and remission of sarcoidosis are often spontaneous in nature, so it will be difficult to maintain whether methotrexate is ameliorating her symptoms or whether Ms. Jones is in spontaneous remission. This was a weakness noted in the critical appraisal. However, given her exacerbation of symptoms with no treatment, it is justified that she continues treatment with methotrexate in lieu of no treatment for the next 18 months if routine lab assessments demonstrate no toxicities. The literature did not comment on the effect of methotrexate on sarcoidosis-related arthralgia, and thus further investigation should be conducted depending on the frequency of Ms. Jones’s joint-related exacerbations, as ibuprofen should not be taken for long periods of time due to potential renal insufficiency. Further, no study has had participants receive methotrexate for more than two years duration, and thus Ms. Jones should be further evaluated at that time for other possible therapies.

## Conclusions

A review of the current literature is often necessary to help guide clinical decision-making for patients with chronic diseases like sarcoidosis. It can be difficult to find studies that match the clinical context of a certain patient in question, as a multitude of social and economic factors come into play when treating the patient, which is much different than strictly treating the disease itself. Patients may not fulfill the exact inclusion criteria of a study, so it can be difficult to apply the results of the study to their situations. Although Ms. Jones fulfilled the inclusion and did not fulfill the exclusion criteria of the critically appraised study, her results as a participant in the study are unknown to us. Thus we could understand that some patients benefit from methotrexate monotherapy, and some do not. Nevertheless, taking into account the patient’s preferences and needs while performing a thorough search of scientific literature for valid, evidence-based conclusions regarding the efficacy of certain therapeutic agents can help a clinician navigate this joint decision-making progress that takes place on a day-to-day basis.

## References

[1] Baughman RP, Winget DB, Lower EE (2000). Methotrexate is steroid sparing in acute sarcoidosis: results of a double blind, randomized trial. Sarcoidosis Vasc Diffuse Lung Dis.

[2] Baughman RP, Lower EE (1990). The effect of corticosteroid or methotrexate therapy on lung lymphocytes and macrophages in sarcoidosis. Am Rev Respir Dis.

[3] Baughman RP, Lower EE (1999). A clinical approach to the use of methotrexate for sarcoidosis. Thorax.

[4] Goljan-Geremek A, Bednarek M, Franczuk M (2014). Methotrexate as a single agent for treating pulmonary sarcoidosis: a single centre real-life prospective study. Pneumonol Alergol Pol.

[5] Ebell MH, Siwek J, Weiss BD, Woolf SH, Susman J, Ewigman B, Bowman M (2004). Strength of recommendation taxonomy (SORT): a patient-centered approach to grading evidence in the medical literature. J Am Board Fam Pract.

